# Evaluation of the Results of Minimally Invasive Plate Osteosynthesis Using a Locking Plate in the Treatment of Distal Femur Fractures

**DOI:** 10.7759/cureus.23617

**Published:** 2022-03-29

**Authors:** Abdelmonem H Abdelmonem, Ahmed Y Saber, Mohamed El Sagheir, Awad El-Malky

**Affiliations:** 1 Trauma and Orthopaedics, West Hertfordshire Hospitals NHS Trust, Watford, GBR; 2 Trauma and Orthopaedics, Calderdale and Huddersfield NHS Foundation Trust, Huddersfield, GBR; 3 Trauma and Orthopaedics, Dumfries and Galloway Royal Infirmary, Dumfries, GBR; 4 Trauma and Orthopaedics, El Hadara University Hospital, Alexandria, EGY

**Keywords:** minimally invasive surgery, locking plate, liss, mipo, distal femur fracture

## Abstract

Introduction

Distal femur fractures are serious injuries that can be difficult to treat, carry an unpredictable prognosis, and lead to long-term disability and morbidity. The introduction of minimally invasive plate osteosynthesis (MIPO) avoids direct exposure of the fracture site, improves fracture healing and decreases the incidence of complications. The aim of this study was to assess prospectively the early results of the treatment of supracondylar fractures of the femur using minimally invasive percutaneous osteosynthesis using a distal femoral locking plate. The study was a prospective study that included 20 adult patients who sustained distal femur fractures.

Materials and methods

The study was a prospective study that included 20 patients suffering from supracondylar fractures of the femur. All patients had fixation of the fracture using a distal femur locking plate (less invasive stabilization system (LISS)) in a minimally invasive technique using an anterolateral or direct lateral approach to the distal femur according to the fracture classification. The follow-up was done using the functional evaluation scale for distal femoral fractures as regards range of motion, deformation, pain, walking ability, and return to work.

Results

The mean age was 52.80 (19-80) years. The mean body mass index of the patients was 28.50, with a range of 23-43 kg/m^2^. The mechanism of trauma was road traffic accidents (RTAs) in nine patients (45%) and falling from standing height in eleven patients (55%). Fractures were classified according to the Arbeitsgemeinschaft Osteosynthesefragen-Orthopedic Trauma Association (AO-OTA) classification. All patients were followed up for a period of six months and assessed in terms of knee range of motion, deformation, pain, walking ability, and return to work. The mean time of radiological union, in which bony trabeculae crossed the fracture gap, was 3.45 ± 0.79 months. The final results obtained were excellent in four patients (20%), good in nine patients (45%), fair in five patients (25%), and poor in two patients (10%). Complications encountered were knee stiffness (20%), superficial wound infection (10%), and shortening (15%).

Conclusion

LISS plating using the MIPO approach is useful in treating complex distal femoral fractures. Large studies from independent centers reporting long-term results are needed to further evaluate the role of LISS plating and the MIPO approach in the management of complex distal femoral fractures.

## Introduction

Distal femur fractures represent between 3% and 6% of all femur fractures [1]. They occur in a bimodal distribution: 15-50 years of age, predominantly in males, sustaining high-energy trauma, and above 50 years of age, predominantly in females, with osteoporosis, who sustain relatively low energy trauma [2]. Problems with conventional open reduction and internal plate fixation of distal femoral fractures are well established. They have been associated with extensile exposures of the fracture site [3].

The introduction of minimally invasive plate osteosynthesis (MIPO) avoids direct exposure of the fracture site, improves fracture healing and decreases the incidence of complications [4,5]. MIPO techniques also decrease time under general anesthesia and blood loss [6].

Less invasive stabilization system (LISS) Synthes plate^TM^ (Synthes, West Chester, PA, USA) was designed for use with minimally invasive approaches and indirect reduction techniques, with the aim of making these difficult goals easier to achieve [7]. Being a locking plate, it also stands off the bone, acts like an ‘‘internal’’ fixator, does not crush the periosteum, and thereby theoretically preserves the blood supply [8].

Although locking plates have provided a valuable additional option for the treatment of distal femur fractures, complications related to slow healing including, non-union, delayed union, and implant failure, are not infrequent and are ongoing problems in managing these fractures [9].

The aim of this study was to evaluate the early results of the treatment of supracondylar fractures of the femur using a less invasive stabilization system (LISS) through a minimally invasive percutaneous osteosynthesis (MIPO) approach.

This article was previously presented as a short free paper at the 41st SICOT orthopedic world congress on September 15, 2021.

## Materials and methods

This study included 20 patients who presented to El Hadara University Hospital, Alexandria, Egypt, after sustaining a supracondylar fracture of the femur. Inclusion criteria included age above 18 years old and a closed supracondylar fracture of the femur. All of the patients were treated with a less invasive stabilization system (LISS) locking plate through the minimally invasive percutaneous approach (MIPO).

Patient demographics, fracture classification, affected side, mechanism of trauma, associated medical conditions, associated injuries, and mean time from injury to the operation were noted.

Fractures were classified according to the Arbeitsgemeinschaft Osteosynthesefragen-Orthopedic Trauma Association (AO-OTA) classification [10]; eight patients had type A1 fracture (40%), one patient had type A2 fracture (5%), five patients had type A3 fracture (25%), one patient had type C1 fracture (5%), and five patients had C2 fracture (25%) (Table 1). Three patients (15%) had associated injuries: the first had an ipsilateral supracondylar fracture humerus that was managed by open reduction and internal fixation using plates and screws; the second patient had an ipsilateral bimalleolar fracture ankle that was managed by open reduction and internal fixation using plates and screws for the lateral malleolus and screws only for the medial malleolus, and the third patient had an ipsilateral tibial plateau Schatzker type I fracture that was managed by pinning under the C-arm. 

**Table 1:** The age of the patients and the fracture pattern as per the AO-OTA classification. The table shows the age of the patients and the fracture pattern, with the comminuted type C2 fracture occurring mainly in those above 65 years of age. AO-OTA: Arbeitsgemeinschaft Osteosynthesefragen-Orthopedic Trauma Association.

Patient number	Age	Fracture pattern
Pt 1	50	A1
Pt 2	51	A1
Pt 3	19	A1
Pt 4	20	A1
Pt 5	55	A1
Pt 6	55	A1
Pt 7	55	A1
Pt 8	45	A1
Pt 9	40	A2
Pt 10	60	A3
Pt 11	62	A3
Pt 12	50	A3
Pt 13	60	A3
Pt 14	58	A3
Pt 15	65	C1
Pt 16	80	C2
Pt 17	55	C2
Pt 18	41	C2
Pt 19	68	C2
Pt 20	67	C2

All patients were assessed clinically and radiologically at the time of presentation, and standard anteroposterior and lateral x-ray views of the distal femur and knee joint were done pre-operatively. In cases of complex multiplane fractures, axial computerized tomography with frontal and sagittal plane reconstruction was done to help in planning the surgical treatment.

All patients were followed up for a period of six months and assessed both clinically and radiologically according to the functional evaluation scale developed by Sanders et al. for distal femoral fractures (Table 2) [11]. The effect of patient- and fracture-related factors on the final functional outcome were statistically analyzed using the t-test, Chi-square test, and Fisher tests.

**Table 2:** The functional evaluation scale developed by Sanders et al. for distal femoral fractures. Excellent: 36-40 points, good: 26-35 points, fair: 16-25 points, and poor: 0-15 points. Sanders et al. [11].

I. Range of motion of the knee joint (in degrees)	Points	Results
Flexion		
>125	6	Excellent
100-124	4	Good
90-99	2	Fair
<90	0	Poor
Extension		
0	3	Excellent
≤5	2	Good
6-10	1	Fair
>10	0	Poor
II. Pain		
None	10	Excellent
Occasional or with changes in weather, or both	7	Good
With fatigue	5	Fair
Constant	0	Poor
III. Deformity		
Angulation (degrees)		
0	3	Excellent
<10	2	Good
10-15	1	Fair
>15	0	Poor
Shortening (cm)		
0	3	Excellent
<1.5	2	Good
1.5-2.5	1	Fair
>2.5	0	Poor
IV. Walking ability		
Walking		
Unrestricted	6	Excellent
30 minutes to <60 minutes	4	Good
<30 minutes	2	Fair
Walks at home, is confined to wheelchair, or is bedridden	0	Poor
Stair climbing		
No limitation	3	Excellent
Holds rail	2	Good
One stair at a time	1	Fair
Elevator only	0	Poor
V. Return to work (either A or B)		
A. Employed before the injury		
Returned to the preinjury job	6	Excellent
Returned to the preinjury job with difficulty	4	Good
Altered full-time job	2	Fair
Part-time job or unemployed	0	Poor
B. Retired before the injury		
Returned to preinjury lifestyle	6	Excellent
Needs occasional help with shopping or laundry	4	Good
Needs assistance at home with activities of daily living	2	Fair
Moved in with family or nursing home	0	Poor

Operative technique

**Figure 1:**
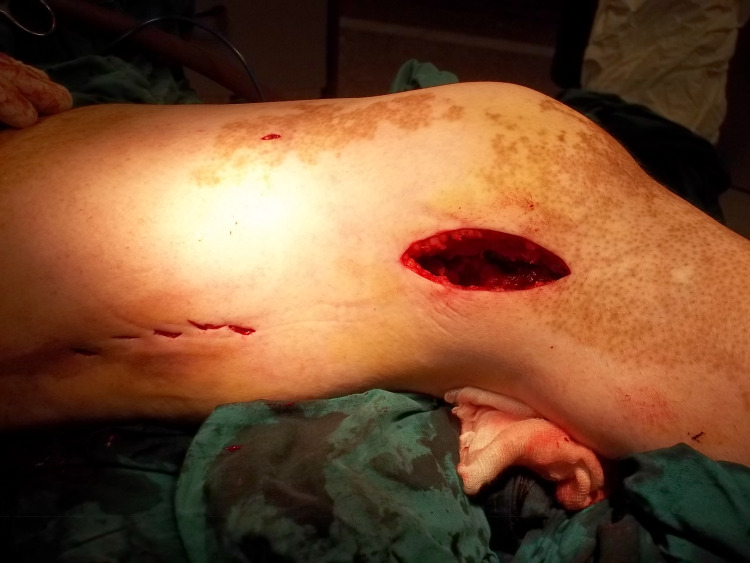
Lateral approach to the distal femur. The patient is supine on the radiolucent table with a bump beneath the ipsilateral buttock. If there is no intra-articular extension, then a distal lateral incision is made starting just proximal to the lateral epicondyle and continuing distally. The incision is sharply continued through the iliotibial band and underlying vastus lateralis in line with their fibers. Dissection proceeds to bone without disruption of the overlying periosteum.

**Figure 2:**
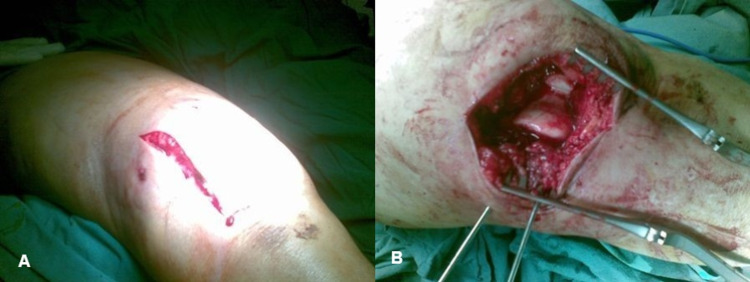
(A) and (B) Anterolateral approach to the distal femur. If there is the intra-articular extension, then a longitudinal skin incision and a lateral parapatellar arthrotomy are done to allow direct visualization of the distal femoral articular surface for anatomic reduction and fixation.

All patients had the surgery under fluoroscopic guidance in a supine position on a radiolucent table. A towel bump was then placed under the ipsilateral buttock to counteract the normal external rotation of the lower limb. A minimally invasive approach was used after the preparation of the affected lower limb. It was either a direct lateral approach if there was no intra-articular extension, as in AO type A (Figure 1), or an anterolateral approach if there was the intra-articular extension, as in AO type C (Figure 2). All procedures were done with percutaneous sliding of the plate through an anterolateral approach in six patients and a direct lateral approach in 14 patients.

Multiple reduction aids such as manual traction, distal femur condyle Schanz pin, and towel bumps under the distal femur were used to overcome the hyperextension deformity of the distal fragment.

**Figure 3:**
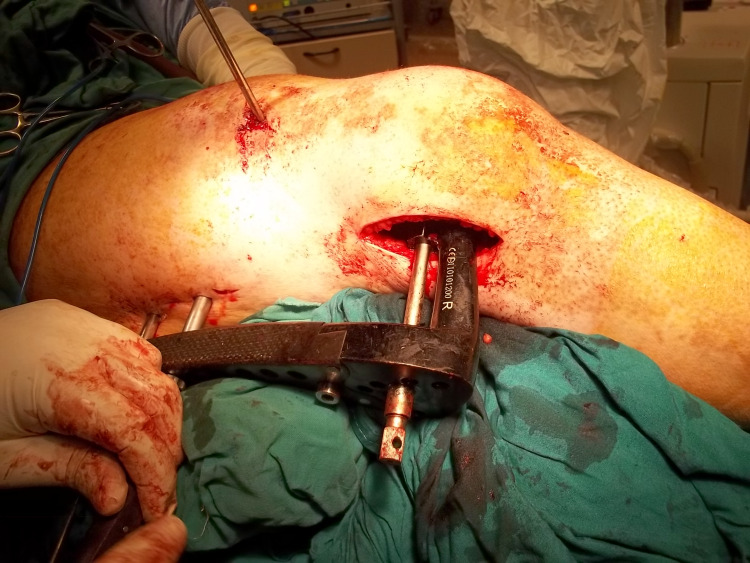
Sub-muscular sliding of the LISS plate. The LISS plate is inserted in a submuscular manner through the distal femur incision, spanning the metaphyseal fracture without direct exposure. Through an incision over the most proximal hole, a proximal connecting bolt was screwed into the proximal end of the plate. A Schanz pin has been inserted through the anterior thigh incision to aid in the reduction of the fracture at the metaphyseal-diaphyseal junction. LISS: less invasive stabilization system.

Fixation was done according to the standard AO principle, starting with the reduction of the articular fragments and fixation with standard cancellous screws, followed by sliding the LISS plate sub-muscularly beneath the vastus lateralis after indirect reduction of the metaphyseal fracture had been achieved (Figure 3).

**Figure 4:**
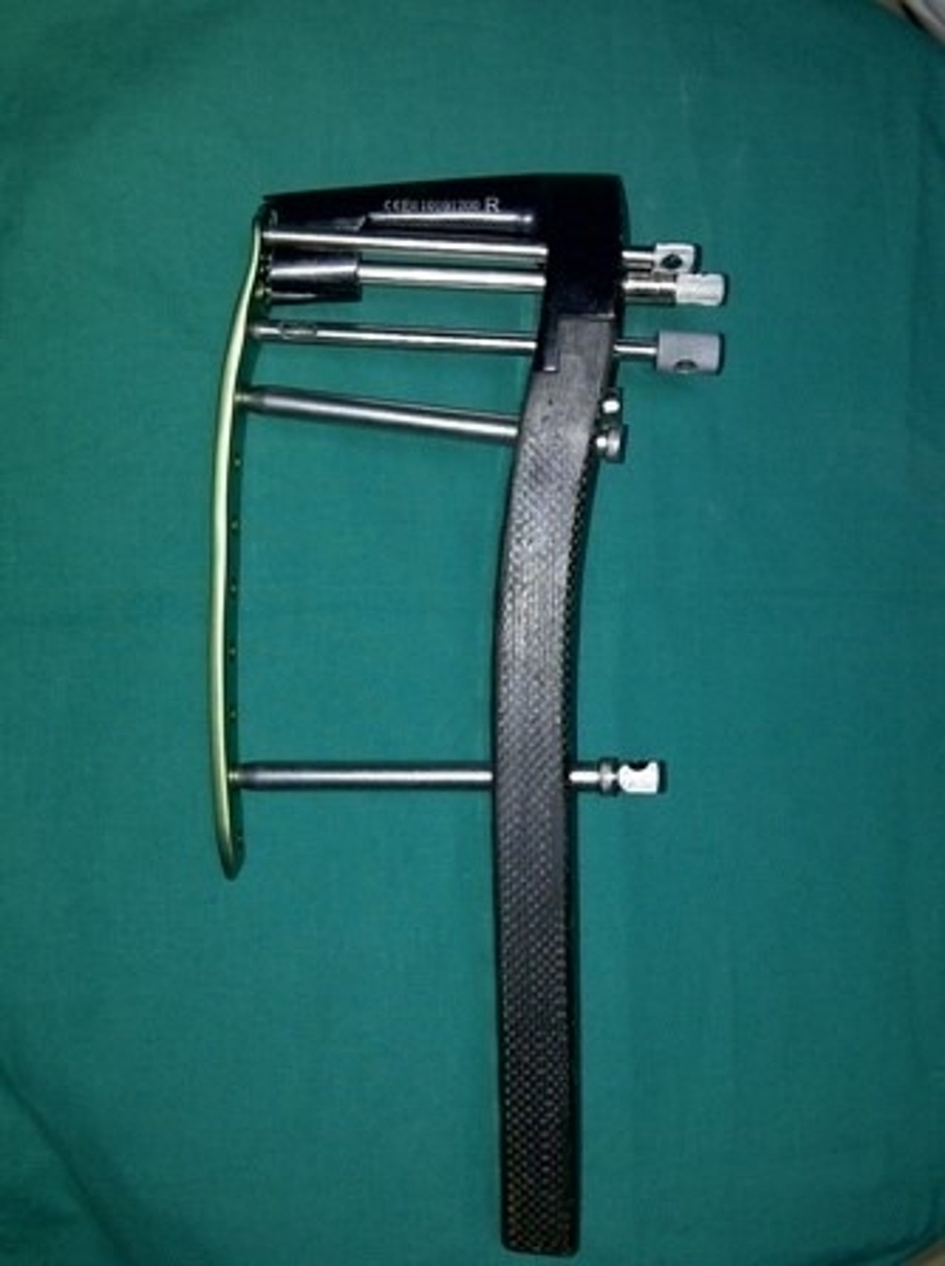
The external jig of the LISS. Assembly of the external jig and the LISS plate. The connecting bolts are screwed through the jig into the distal and proximal parts of the plate to allow for the insertion of the screws using small incisions. LISS: less invasive stabilization system.

**Figure 5:**
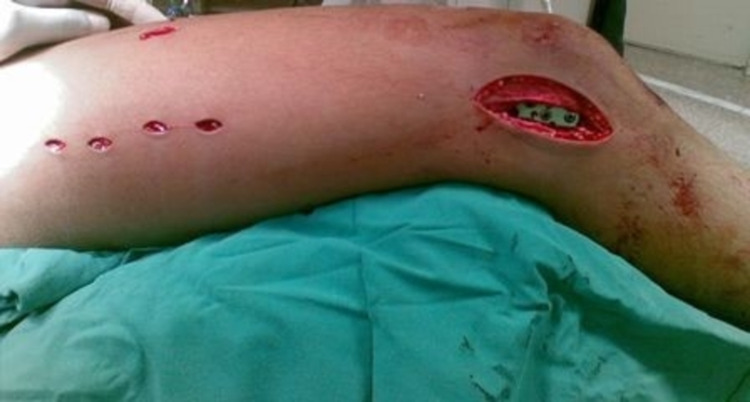
Proximal screws are inserted through multiple small incisions. Postoperative photo demonstrating incisions after the implant has been placed. The lateral distal incision for the insertion of the plate and distal screws, and multiple small proximal incisions for the insertion of the proximal screws.

After the insertion of the distal screws, the proximal screws were inserted using the external jig through multiple small incisions (Figure 4 and Figure 5). 

**Figure 6:**
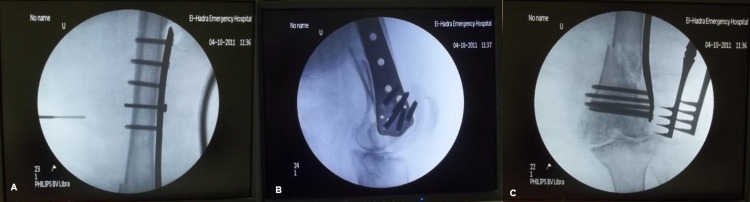
Intra-operative fluoroscopy images. Images show that fluoroscopy is used intra-operatively to check the appropriate length and position of the plate and check the reduction and alignment of the fracture in both antero-posterior and lateral views.

The fracture reduction and the implant position are checked under fluoroscopic guidance (Figure 6A-6C).

All wounds were copiously irrigated. The joint capsule and iliotibial band were closed utilizing absorbable sutures, followed by the closure of subcutaneous tissue and the skin.

Postoperative protocol

Postoperative radiographs were obtained to check the reduction and adequacy of the fixation. Exercises started from the first postoperative day in the form of passive and assisted active flexion and extension range of motion exercises of the knee joint, straight leg raising if tolerated by the patient and quadriceps strengthening exercises. Patients were discharged in a hinged knee brace with a sequential increase in the range of flexion of 30 degrees every two weeks. All patients were non-weight-bearing for eight weeks, followed by partial weight-bearing for two weeks, and then full-weight-bearing afterward.

Stitches were removed two weeks postoperatively. The patient examination and radiological evaluation were carried out after six weeks, three months, and six months.

Case example

**Figure 7:**
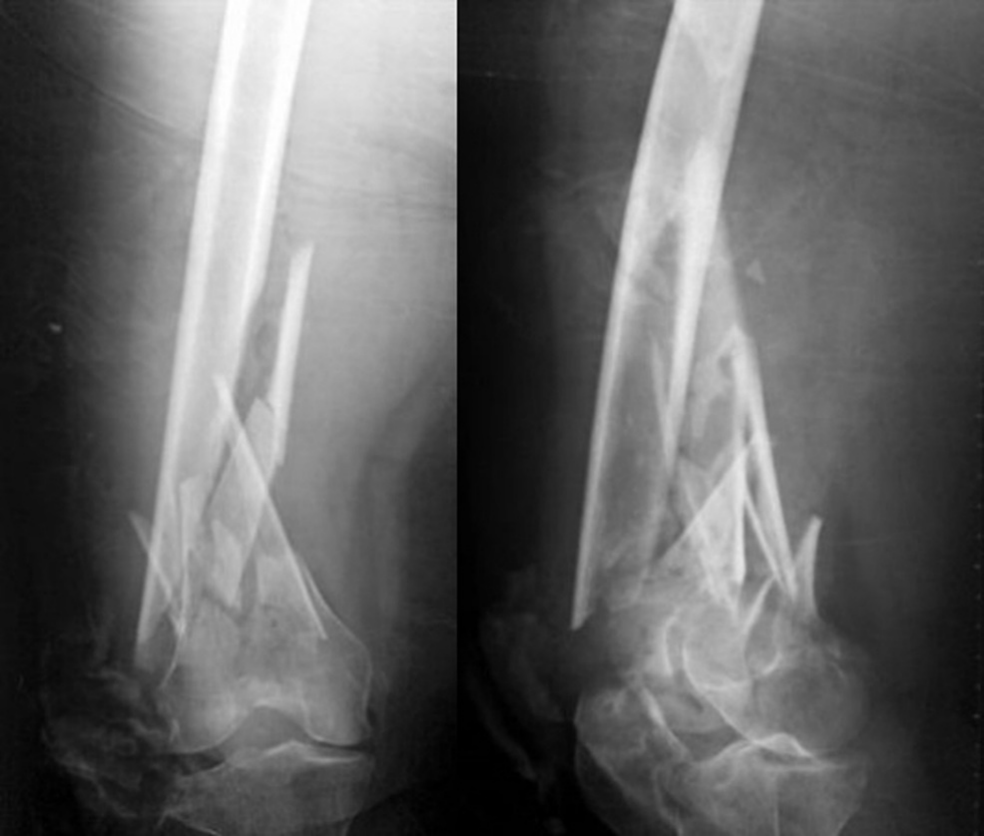
Pre-operative x-rays. X-rays show a comminuted (AO type C2) supra-condylar femur fracture with intra-articular extension.

**Figure 8:**
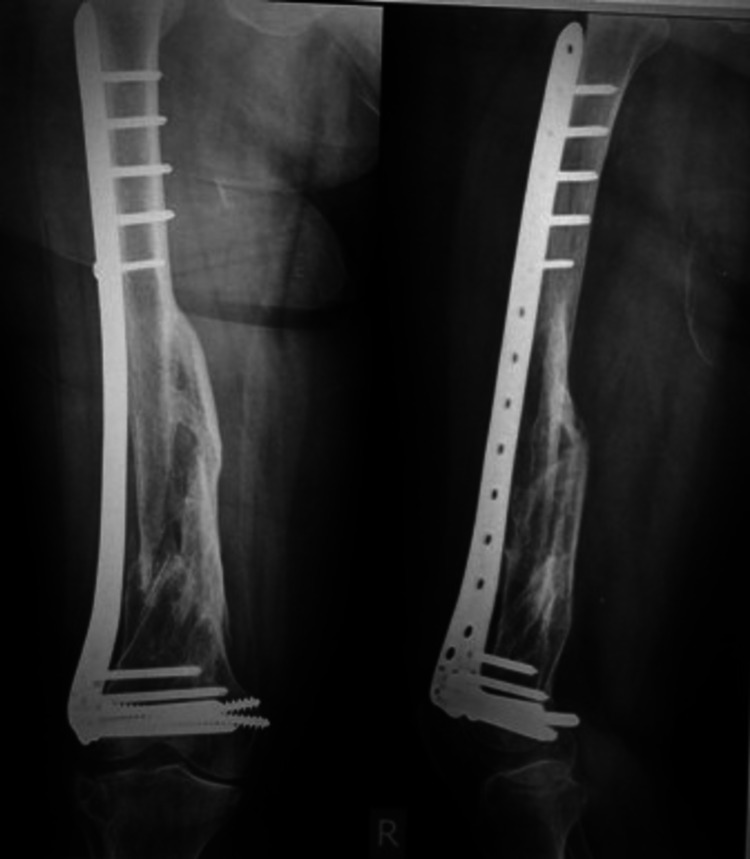
Six months post-operative x-rays showing bridging callus formation. Six months follow-up x-rays show bridging callus with satisfactory alignment in both antero-posterior and lateral views with no signs of implant failure.

An 80-year-old female sustained a closed intra-articular (AO type C2) supracondylar fracture of her right femur after a road traffic accident. Surgery was done 11 days after the injury, after the patient's medical condition was optimized. Union was achieved at three months postoperatively when she started weight-bearing. At six months, she achieved a (good) functional outcome score of 29 points with more than 100° of knee flexion. Pre-operative and six-month post-operative x-rays are shown in Figure 7 and Figure 8.

## Results

Six patients were male (30%) and 14 patients were female (70%). The mean age was 52.80 ± 14.79 (19-80) years. The right side was affected in 12 patients (60%) and the left side in eight patients (40%). The mean body mass index of the patients was 28.50, with a range of 23-43 kg/m^2^. Fourteen patients were housewives (70%), four were manual workers (20%), and two were clerks (10%). Eleven patients (55%) had associated medical conditions. Four patients had hypertension, two patients suffered from ischemic heart disease, two patients had uncontrolled diabetes, two patients had both diabetes and hypertension, and one patient was asthmatic. The mechanism of trauma was road traffic accidents (RTAs) in nine patients (45%) and falling at home in eleven patients (55%).

According to the Sanders functional evaluation scale, the mean score was 26.7 ± 8.5 SD. The results obtained after six months of follow-up were excellent in four patients (20%), good in nine patients (45%), fair in five patients (25%), and poor in two patients (10%) (Table 3). 

**Table 3:** Distribution of the studied sample patients regarding the net functional outcome.

Functional outcome	Number of patients	Percentage %
Poor	2	10
Fair	5	25
Good	9	45
Excellent	4	20

The mean time of radiological union, in which bony trabeculae crossed the fracture gap, was 3.45 ± 0.79 months (range 2.5-5.5 months). About 45% of the patients achieved radiological union within three months, 40% within four months, and 15% more than four months.

The younger the age of the patients, the better the results were. However, the relationship between age and the final score was statistically insignificant (p-value 0.224). All patients with an excellent final score were below the age of 60, and 77% of patients with good final scores were between the ages of 40 and 60. All patients with an excellent outcome and 44% of patients with a good outcome had a BMI below 30 kg/m^2^. The two patients with a poor final score were above 30 kg/m^2^, but this relationship was statistically insignificant (p-value 0.093).

About 75% of patients who achieved excellent results had no associated injuries, while 25% of them had associated injuries. Three patients had associated injuries; one patient achieved an excellent final score, and two patients achieved a good score. However, these differences were statistically insignificant (p-value 0.672). 

The minimum time lapse before surgery was one day and the maximum was 14 days, with a mean of 4.6 days. However, there was no statistically significant relationship between the time-lapse before the operation and the final outcome (p-value 0.819).

Eleven patients did not have any complications during the follow-up period. Knee stiffness with a limited range of flexion of varying degrees occurred in four patients (20%). Two patients had a type C2 supracondylar fracture of the femur, one patient had a type A1 and one patient had a type A3 fracture. It was noticed that stiffness prevailed in intra-articular fractures and was aggravated by the increase in the degree of comminution, prolonged delay of surgery, extended periods of knee immobilization, and the delay of physiotherapy, which allowed for more intra-articular adhesions to be formed.

Three patients (15%) had a shortening of more than 2 cm, but two of them achieved a fair final score. Shortening occurred in comminuted type C2 fractures where leg length had to be sacrificed to ensure bone continuity and fracture stability. Malalignment was encountered in this series in seven patients (35%), but all of them were less than 10 degrees of angulation.

Superficial wound infection occurred in two patients (10%). The first was a 60-year-old diabetic female with poorly controlled diabetes who had an AO type A3 supracondylar fracture. Surgery was delayed for three days for control of diabetes. The second patient was a 55-year-old diabetic female who had an AO type C2 supracondylar fracture. Surgery was done two days after the trauma. The superficial infection settled down with two weeks of oral antibiotics in both cases, with no further wound complications. No cases in the study showed implant loosening or breakage during the duration of follow-up.

## Discussion

Distal femoral fractures result from two principal mechanisms of injury. These are either high energy trauma, such as road traffic accidents, which may be open injuries with considerable comminution of the condyles and metaphysis, or low energy trauma, in elderly populations with severe osteoporosis, which may be further complicated by the involvement of total knee arthroplasty as periprosthetic fractures [12]. 

The goals for treatment of distal femur fractures are the restoration of bone length, anatomic articular surface alignment and rotation, early mobilization of the associated joints, rapid fracture union and minimal complications [4].

Many different fixation methods have been described. Fixation options can be grouped into three broad categories: open anatomical reduction with plate and screw fixation, bridge plating or submuscular plating, and retrograde intramedullary nailing [13].

Biomechanical studies have suggested that when condylar buttress plates and dynamic condylar screws have been compared to the LISS system, the LISS fixation system was able to withstand higher loads, providing more stable fixation than conventional implants [14]. A comparative study between intramedullary nails and LISS in the management of extra-articular supracondylar femur fractures found that nailing proved more cumbersome intraoperatively due to escalated operating time and blood loss and successive anterior knee pain necessitating implant removal, but this detriment may be offset by an inclination toward earlier union [15]. 

Biological fixation techniques have been developed in an effort to lessen the incidence of surgical complications in lower-extremity fractures by minimizing the amount of surgical trauma, thus sparing the remaining vascular supply and the tropic factors in the fracture hematoma [16].

Fixation using the LISS and the MIPO technique is successful in extra-articular AO A1-A3 metaphyseal fractures as well as intra-articular displaced C1-C3 fractures, which can be managed with open reduction of the articular surface and MIPO plating of the metaphyseal fracture. Metaphyseal fractures above a stable total knee prosthesis or below a well-fixed total hip prosthesis are also typically amenable to indirect reduction and MIPO fixation [17]. Better outcomes were observed when longer plates were used, especially in complex articular fractures (type C) in the elderly [18].

This study is a consecutive prospective study including 20 patients with high and low energy type A and C distal femoral fractures (based on the AO classification), and the results obtained were found to be comparable to the results of other studies that used the same method of treatment.

In this study, the mean period to fracture union was 3.45 ± 0.79 months (range 2.5-5.5 months) given that no primary cancellous bone graft was used in any of the cases. All fractures healed without the need for secondary bone grafting. In total, one case needed secondary procedures including wound debridement and irrigation for control of infection. There were no cases of failed fixation or implant breakage.

Fankhauser et al. treated 30 distal femoral fractures (types A and C) using the LISS, and patients were followed up for a mean period of 20 months. They reported a mean period of union of 12 weeks with a range of 8-23 weeks, and the time till full-weight-bearing ranged from 6 to 18 weeks [13].

Weight et al. used the LISS to treat 22 distal femoral fractures, and patients were evaluated at an average of 19 months post-injury. All fractures healed without secondary surgeries at a mean of 13 weeks (range 7-16 weeks). There were no cases of failed fixation, implant breakage, or infection [19]. 

Kanabar et al. reviewed records of six men and 11 women who underwent LISS plating for complex distal femora fractures. The mean time to union was 17 weeks. Two patients with non-union underwent a second LISS plating and bone grafting, resulting in a satisfactory final outcome. Delayed radiographic union was observed in one patient, but clinically, he was asymptomatic and mobile. The fracture finally united at nine months [4]. 

This study showed that the younger the age of the patients, the better the results were. The mean age of the patients with excellent results was 35, and the mean age of the patients with good results was 55. However, the relation between age and the final score was statistically insignificant. Likewise, Schütz looked at the relationship between fracture type, patient’s age, mechanism of trauma, type of reduction, or soft-tissue injuries and the outcome parameters that he measured (fracture healing, weight-bearing, axial relationships, and range of motion) and found no statistical significance to it [20].

Shortening in this series occurred in three patients (15%) who had a shortening of more than 2 cm. Two patients achieved fair final scores, and one patient achieved poor final scores. The first had type A1, the second had type A3, and the last had type C2 supracondylar fracture femur with a maximum difference in leg length of 2.5 cm. In both patients with type A supracondylar femur fractures, which were in the first five cases of the series where the learning curve of the technique was at its beginning, shortening occurred in comminuted type C2 fractures where leg length had to be sacrificed to ensure bone continuity and fracture stability. Malalignment was encountered in this series in seven patients (35%), but all of them were less than 10 degrees of angulation. Schütz et al. reported an axial deviation of 5-10 degrees in 13 cases (20%) [20]. Kanabar et al. reported two patients (12%) with angular malalignment of 10º to 15º [4].

In this series, superficial infection occurred in two patients (10%). Both of them were uncontrolled diabetic patients. Kolb et al. found that the infection rate of the LISS was 3% and the compression plating with the MIPO technique of 2.3%, which is less than the 6% infection rate seen with conventional plate osteosynthesis [21]. In the series done by Markmiller et al. using the LISS and distal femoral nail for treatment of distal femoral fractures, infection occurred in one of the 19 fractures (5.3%) treated with the distal femoral nail. Generally, it is noticed that infection rates can be diminished using newly developed minimally invasive techniques [22]. 

Implant loosening did not occur in this series. Implant loosening is a rare complication with locking plates and it is mainly seen as proximal failure due to eccentric placement of the plate on the lateral femur, which may result in an inadequate purchase of the unicortical screws. In this series, all the proximal screws were bicortical. Weight et al. had no cases of fixation failure or implant breakage, and no fractures required bone grafting [19].

Schandelmaier et al. found four patients whose fixation failed due to proximal screw pullout [12]. Different causes of implant failure were reported, including early-onset weight-bearing, implant length too short, improper implant placement, and insufficient fixation of the femoral shaft with monocortical screws [14,23]. It often occurs in older patients and patients with osteoporosis or periprosthetic fractures who are prone to other complications as well [24].

The limitations of this study include the small number of patients, short-term follow-up, lack of a control group of patients in whom fractures of the same pattern were treated with conventional open reduction technique, and the inclusion criteria did not pertain to a specific type of supracondylar fracture (high or low energy, types A or C fracture). The points of strength of this study include being a prospective consecutive study and the results being measured in terms of functional outcome, not just radiological union.

## Conclusions

LISS plating using the MIPO approach is successful in treating complex distal femoral fractures. Bicortical screws give better fixation and are recommended for osteoporotic patients. Reduced blood loss, low infection rates, and early mobility due to the primary stability of the construct are advantages of LISS plating and the MIPO approach. Large studies from independent centers reporting long-term results are needed to further evaluate the role of LISS plating and the MIPO approach in the management of complex distal femoral fractures.
